# Vaccination in the Light of the Royal British Commission

**Published:** 1899-11

**Authors:** M. R. Leverson

**Affiliations:** Fort Hamilton, L. I., N. Y.


### VACCINATION IN THE LIGHT OF THE ROYAL BRITISH COMMISSION.

M. R. Leverson, M. D., Fort Hamilton, L. I., N. Y.

(Continued from Oct. No., p. 458.)

Q. 4843 (Mr. Meadows White). Woodville speaks of a pustule? Yes; *Jenner having constantly spoken of pustules when he meant ulcers.*

Q. 4844-5. In Woodville's cases an ulcer was only once produced. In the other cases it produced a whitish vesicle like the vaccine vesicle we now see on the arms of children and others. Ulceration frequently followed the use of Woodville's lymph in other hands.

Q. 4848-9. A connection between cow-pox and small-pox was attempted to be proved by Jenner before he published his *Inquiry*, and by his contemporaries within the first few months of the introduction of the practice, first by experiment and then by observing what followed in the vaccinated in the ordinary way of experience.

Q. 4850. The question of the chairman, "What were the experiments?" leads to an exposition of the remarkable self-deception on the part of the vaccinationists which led them to believe they had proved cow-pox to be prophylactic against small-pox. This demonstration was especially unpalatable to the majority of the commission, most of whom had over and over again publicly committed themselves, not merely to the doctrine of the prophylaxy of cow-pox but to its identity with small-pox and to the rigid demonstrations of Jenner and his successors. Accordingly, they not only pursued Dr. Creighton with a severe cross-examination, but (probably unintentionally), made repeated efforts to lead him away from important points into side issues. The result was a valuable discussion, but one which needs to be closely studied to disentangle it from the confusion in which it was involved by the indiscriminate and disconnected questionings of Dr. Creighton by the chairman, by Sir James Paget, by Prof. Michael Foster, by Mr. Savory, by Dr. Bristow, and by Mr. Hutchinson.

Dr. Collins evidently had a clear insight into the whole subject, and with the aid of a pertinent question now and then from him, Dr. Creighton steered his evidence through the rocks and shoals of irrelevancy into clear water. To give an abstract of his testimony in the order of questioning which took place, would weary even an attentive reader. I will try to give its substance, giving the words of the parties to this colloquy only where it seems specially desirable so to do. The reasons before assigned for giving the exact words of the witness have but slight application to the case of those (the opponents of vaccination), whose statements even a prejudiced reader is hardly likely to accuse me of misrepresenting.

Dr. Creighton explained the Suttonian method of inoculation in which the object sought was to produce a single local variolous pustule. Sometimes it produced a general eruption, but frequently nothing but a local pustule, and when this occurred the patient was said to have had small-pox and to be proof against a second attack.* Sutton's inoculation consisted not in inoculating actual small-pox, but matter obtained from a mild inoculated case at an early stage, and so from arm to arm, by which means the effects were reduced "to the mere formality of small-pox; that is to say, to a local pustule and little else" (Qs. 4850 and 4895). Persons who had been infected with the cow-pox, whether by accident or by intentional inoculation, were then subjected to what was called the "variolous test," and when what was produced

* It has been pointed out above that there is no evidence to establish the theory of the auto-protection of small-pox. I am sorry to observe that Dr. Creighton adopts the current view (Q. 5003). He gives no reason for this opinion; but there was no call for him to do so at that time, therefore the omission here noted is not intended as a reflection upon him. —M. R. L.

was the same as the result of the Suttonian inoculation, he was said to have resisted the small-pox. Dr. Creighton says (Q. 4852), "It appears to me that the margin of difference (between the results of the variolous tests following upon vaccination, and the ordinary result of the Suttonian inoculation) *was an imperceptible one; that practically the same results followed*. In the hope of diminishing the effect of this accurate detection of the variolous test fallacy which if not got rid of, it was seen, utterly destroyed every appearance of scientific support on which vaccination has been supposed to rest, Dr. Creighton was specially examined on the case of the boy Phipps, concerning whose vaccination and subsequent sub--mission to "the variolous test," Mr. Savory stated Jenner said, "The boy has since been inoculated for small-pox which as I ventured to predict, produced no effect." But this was in a letter to his friend Gardiner, and Dr. Creighton calls it Jenner's colloquialism (Q. 4862), contrasts it with his statement in the *Inquiry* in which a formula is used similar to what he uses in a letter to Shorter, of Bloxham, and which in that case clearly meant *a local pustule*, and says, "I would attach no importance to slight differences in Jenner's language, because he was notoriously unprecise in his statements (Q. 4864). Dr. Creighton calls attention to Jenner's discrepant statements, that he was capable of making no difference between 'no effect' and a local pustule, that in the case of Mary James, he admits that the variolous test produced a local pustule, fever, and a transient eruption on the wrist; and when the chairman seeks to get rid of that as just 'one particular case,' Dr. Creighton shows that all Jenner's tests amounted to only two or three cases of children" (Q. 4869). Woodville recorded some 400 cases, all of whom had been vaccinated, but many of these were attacked by small-pox and the variolous test was applied to all indiscriminately. Woodville took the small-pox cases to be the results of his cow-pox inoculations, and biased unwittingly by the impudent title of "small-pox of the cow," given to cow-pox by Jenner, he concluded from these facts, that cow-pox was indeed derived from human small-pox, and this belief was widely spread before the fact was discovered that Woodville's cases were valueless to the end to which they were quoted, because of the fact that he vaccinated in the small-pox laden atmosphere of the small-pox hospital. To these small-pox cases, as well as to those of cow-pox, he applied "the variolous test." Woodville never drew any contrast between the results of the ordinary Suttonian inoculations and the results of the variolous test in these cases. Woodville's cases having spread the erroneous notion of the identity of cow-pox and small-pox, the belief continues to this day, in spite of the discrediting of those cases.

"The sequence of events," according to Dr. Creighton (Q. 4914), "was this, that when they inoculated a cow-poxed subject and found only very little effect they said 'It does not signify,' forgetting that very shortly before they had been content with that same effect as a due result of small-pox inoculation."

The lightness of the effects of the Suttonian inoculation may be judged from the criticism of Salmade (Q. 4912), a French physician, who wrote a treatise on inoculation about the same time as Jenner's *Inquiry* was published. In this he stated that after taking the matter from arm to arm in what the Suttons called a crude state (*i. e.*, in an early stage of the pustule) through a certain number of arms, the effect would go off altogether.

This was, nevertheless, supposed to be a sufficient attack of small-pox to protect from a future attack; but when applied after a cow-pox inoculation and an effect was produced with a "margin of difference absolutely imperceptible" from the Suttonian effect, it was recorded as proof that the subject was no longer susceptible by reason of his cow-poxing. The majority of the Commission were absolutely staggered by this discovery, and tried over and over again to destroy this seemingly incredible fact, but Dr. Creighton piled evidence upon evidence in its proof. In the view taken by the majority of the medical profession, *and in which Dr. Creighton concurs,* that small-pox is auto-protective, the detection of this remarkable fallacy would be fatal to the pretension that cow-pox is bovine small-pox, for if small-pox can be taken so immediately after it, cow-pox must be something different from it. The editor of this abstract does not share in the prevailing opinion of the auto-protection of small-pox. To him the fact that a state of things being regarded as one thing when it was desired to be that thing, and as nothing at all when it was desired to be nothing, furnishes to him only a fresh illustration of the marvelous influence of *bias* or *prejudice* upon otherwise intelligent men to hinder the perceiving of palpable truths, and to cause things to be imagined as seen which have no existence in fact.

Dr. Creighton disposes of a criticism made upon the summary of the Ward cases, given on pp. 130-31 of his work, *Jenner and Vaccination*, by supplying a copy of Mr. Ward's statement from the *Medical and Physical Journal*, II, p. 134, September, 1799. In his summary, Dr. Creighton stated Mr. Ward's Case 1 is one of inoculated small-pox following vaccination. It turns out to be really a case either of *in*-vaccinated small-pox, or of concurrent cow-pox inoculation and small-pox. Cases 2, 10, and 11 of Mr. Ward's report seem to be of a similar kind. It is evident that Mr. Ward thought the small-pox which supervened upon the inoculation to be a normal effect of cow-pox inoculation, and thus helped to spread the illusion of the identity of cow-pox and small-pox. Dr. Creighton has been unable to ascertain the source of Ward's vaccine matter.*

---

*The editor of this abstract has also tried to ascertain the source of Mr. Ward's virus, but without success. It is extremely probable that Mr. Ward was one of the numerous practitioners to whom Woodville distributed what he supposed was cow-pox virus, but which in many cases was small-pox.

Dr. Creighton is of opinion that Case 2 was *caught* from Case I, and not from its being *in*vaccinated, because, while being the brother of Case I and living in the same house, the small-pox did not declare itself until the thirty-third day. "If it had been the vaccine exanthem the difference would have come out sooner" (Q. 4947).

Dr. Creighton sets out all that is important of a published correspondence between Mr. Shorter and Jenner. Mr. Shorter describes the inoculation of Wm. and Ann Barrett, on the 7th of December, 1799 (Q. 4978), "with matter on a thread, taken only the day before. They soon were sensible of some effect from it. The first twenty-four hours they felt smarting and itching in the incisions. On removing the threads they observed them moist. There *was* little further to be perceived until the fourth day, when inflammation was evident on William's arm, sufficient for me to have assured him *that if he had not had the cow-pox I should have been positive he had received the infection.* Ann's arm appeared very doubtful. From this day to the seventh the inflammation was progressive, and on examining the arms *a pustule presented itself on Ann's*, with matter in it on the inoculated part. She also informed me that this day she was very unwell, was feverish and had a tendency to sickness. William's arm was also more inflamed on the fourth day. The incision had a rising appearance, and felt hard, and toward the lower part there was a pustule just appearing; but he had not the least indisposition this day or any succeeding one. On the 9th William called on me and informed me that on the 8th he had observed matter in the pustule on his arm; but when I inspected it this day it had the usual aspect of small-pox just declining. He also told me that the pustule on Ann's had discharged matter on the eighth day, but that on this day it appeared to be dying, and all indisposition gone. On the twelfth day I saw them both again. Ann's arm had inflammation which extended almost from the shoulder to the elbow, and so painful and sore that she applied an emollient poultice to it. The incision now had a large scab upon it, and there was on the bend of the arm a sort of blighted pustule. Ruminating on these appearances for a moment, I could not but persuade myself that she had certainly received the variolous infection."

At Q. 4983 Dr. Creighton gives an extract from Jenner's reply, which is dated 29th December, 1799. ". . . However, I must at once observe that the cases you adduce, in my opinion, do not in the least militate against the safety of cow-pox as a protective against small-pox. Pray, recollect how seldom we find the skin insensible to the action of variolous matter in those who have previously gone through the small-pox. The cow-pox leaves it in the same state. The patients you mention were not insensible to the local action of the variolous virus." *

"This," says Dr. Creighton, "seems to me to explain the periphrasis with which Jenner describes the result of the variolous test upon his first case, the case of James Phipps.

Dr. Creighton also says, in reply to Professor Michael Foster (Q. 4996), "I can confidently say that there is no distinction drawn by any one between the local pustule and the proper pustule," but (Q. 5000) he agrees with Sir James Paget "that the variolous test produces the same effect after vaccination in Jenner's cases as after small-pox."

At Q. 5008, Prof. M. Foster and Dr. Bristow established the fact that after vaccination the results of inoculation were generally mild. But Dr. Creighton shows that the ordinary Suttonian inoculation was also frequently so.

Dr. Creighton then puts in evidence two control experiments, one conducted by Ballhorn and Stromeyer, and the other by Sacco. On the Chairman asking if Drs. Ballhorn

---

* William Cobbett's saying in relation to Jenner will doubtless recur to the reader: "Quackery has always one shuffle left."

and Stromeyer were medical men of any repute (Q. 5010), Dr. Creighton informs him, "They were indeed; they were the leaders of the vaccination movement in Germany."

(Q. 5017.) "But apart from their being leaders of the vaccination movement, were they medical men of any repute? Yes, undoubtedly; one was a court surgeon, and the other was, I think, a court physician at Hanover."

Two children of a Mr. Kirchner were vaccinated on June 26th, 1799 (Q. 5019). That on the boy succeeded; that on the girl is stated to have failed. On April 24th, 1800, both brother and sister were variolated. The result in both is practically the same. "On the 28th the place where the blister had been placed upon the two children had suppurated" (Q. 5021). The small-pox poison had been placed in a blister intentionally produced upon the left arm of each of them, and yet in the face of a description of each case, showing that "the margin of difference was an imperceptible one; that practically the same results followed." Drs. Ballhorn and Stromeyer summed up these cases thus: "The whole of this history proves that the inoculation of small-pox *had no effect upon the boy*" (the inoculated spot suppurated *on both!*), "and that the small-pox of his sister was extremely benign and mild; which might be a beneficent result of the trial of the vaccine inoculation, even where its success has been null or incomplete." *

Dr. Creighton in answer to Mr. Meadows White (Q. 5047), gives the cases of Thornton, of Strand, in which the variolous test failed. These cases were published by Dr. Beddoes in his contributions to medical knowledge in 1799. Mr. Savory at Qs. 5050-1-2, tried hard to excuse these failures on the ground that at that early stage it was natural that errors as to the time of taking the lymph should be made.

---

* How remarkable and striking an illustration is here seen of the inveteracy of prejudice and bias, leading such men as Drs. Ballhorn and Stromeyer to see what they want to see, in the teeth of contrary facts stated by themselves!

